# Supplementary material for: Are carbon emissions trading and green financial instruments synergistic? -Comprehensive quantitative research based on content analysis
Source: PLoS One. 2024 Mar 7;19(3):e0298601. doi: 10.1371/journal.pone.0298601 (PMC10919601; doi:10.1371/journal.pone.0298601)
Supplement: S1 Data — (ZIP) [file pone.0298601.s001.zip › Data/地级市Z_F工作报告.zip!/╦─┤¿/╫╩╤⌠╩╨/└┤╫╘╣1⁄2╓┌║┼í╛┬φ┐╦╩2╛▌═°í┐_2018.docx]

各位代表：

现在，我代表市人民政府向大会报告工作，请予审查，并请市政协委员和其他列席人员提出意见。

2017年工作回顾

2017年，在省委、省政府和市委坚强领导下，在市人大、市政协监督支持下，我们紧紧依靠全市人民，始终保持专注发展定力，迎难而上、拼搏奋进，完成地区生产总值1022.2亿元、增长7.8%，全社会固定资产投资594.1亿元，规模以上工业增加值增长9.8%，地方一般公共预算收入49.8亿元、同口径增长8.7%，社会消费品零售总额365.7亿元、增长12.2%，城乡居民人均可支配收入分别增长8.3%、9.3%，PM2.5、PM10浓度分别下降23.8%、10.8%，各项工作取得新的成绩。

过去的一年，是极不平凡的一年，面对诸多大事要事难事，我们强力攻坚，推动了一批重点难点工作取得突破：经济总量在区划调整后再次迈上千亿台阶，资阳发展站上新的起点；成资一体化轨道交通资阳线、成资大道、“张老引水”工程三大标志性项目取得实质性进展，临空经济区和中国牙谷建设高点起步，为资阳未来发展奠定了坚实基础；凤岭公园等一批城市改造完善提升项目即将竣工，盘踞沱一桥头、文化路多年的“钉子楼”依法拆除，城市面貌明显改观；一批环保突出问题有效整治，城区空气质量改善率居全省第2位，沱江出境断面水质优于入境断面，生态环境质量明显改善；脱贫攻坚连战连胜，义务教育均衡发展取得历史性突破，与省部级龙头医院深度合作带动医疗服务水平大幅提升，群众获得感幸福感明显增强；化解金融风险成效明显，退出全省信用风险重点关注地区，金融生态环境全省排名上升7位；临江寺豆瓣恢复生产，华通柠檬破产重整成功，禾邦集团瘦身减债重组取得突破，四海集团依法重整平稳推进，国际商贸城完成承债式并购，毛地拍卖、问题楼盘有序处置，一批矛盾问题得到有效化解；实现到省进京“零集访”、较大以上安全生产事故“零发生”、防灾减灾“零死亡”目标，社会大局和谐稳定。这一系列重点难点工作，检验了各级干部的能力和作风，回应了广大群众的关切和期盼，坚定了我们继续攻坚克难的信心决心。

一年来，我们坚持稳增长促转型，经济总量质量同步提升。“项目年”活动扎实推进，创新项目推进机制，177个市级重点项目完成投资315.2亿元；新签约落地PPP项目15个、总投资92.6亿元；争取到位中省预算内投资112.9亿元，争取年度用地指标914公顷；策划储备市级重点项目390个、投资总额6900亿元，为未来投资增长做实了项目支撑。制造业加快发展，与省交投集团合作发展新制式轨道交通产业，空铁列车获得商业试用订单，“资阳造”机车累计出口25个国家和地区、总量达800余台，现代“致道”、南骏“瑞吉”商用车成功推向市场，资阳天府·华为云计算中心成功上线；与川投集团联合投资建设运营管理中国牙谷，美国爱齐等28户口腔装备材料企业入驻，首批“资阳造”口腔产品投放市场；安井食品等33个项目有序推进，新增规模企业25户。服务业提质发展，国、省级电子商务试点示范加快推进，电子商务交易额居全省第4位；城乡物流速递网络实现全覆盖；国家级旅游业改革创新先行区建设通过中期评估验收，旅游收入162亿元、增长15.3%；农商行挂牌开业，全市新增贷款76亿元、是上年的4.3倍，新增中小企业贷款41.6亿元。现代农业稳步发展，严格耕地保护，粮食总产量163.8万吨；安岳柠檬产值突破100亿元，温氏生猪一体化项目启动建设，安岳获准创建全国有机产品认证示范区，乐至成功创建全省首个国家级林业科技示范园、获准创建国家农村产业融合发展示范园；“资味”农业品牌体系加快完善，新增认证绿色食品6个、有机食品7个。质量效益持续提升，三次产业结构进一步优化，第三产业占比提高5.7个百分点；财政收入、城乡居民收入稳定增长；民营经济占地区生产总值的56.7%；雁江区跻身全省县域经济综合评价第15位，连续7年荣获“类区先进县”称号。

一年来，我们坚持打基础优环境，城乡发展条件持续改善。城镇建设有力推进，完成第二轮土地利用总规调整，新增建设用地规模47平方公里；城市总规修编基本完成，中国牙谷、临空经济区总体规划和产业规划形成初步成果。成渝客专资阳北站站前广场等项目竣工投用，皇龙片区道路等“七位一体”改造基本完成，沱西滨江景观带等工程即将竣工，娇子大道西延线等项目加快推进，城东14公里滨江路堤景观带全线贯通，临空经济区首批11个项目全面展开。首批80条背街小巷完成整治，惠及20余万市民。中心城区11个农贸市场改造全面完成，拆除违法建设近30万平方米，扬尘污染、油烟噪音、广告乱贴等得到有效遏制。安岳、乐至城市功能不断完善，龙台镇入选国家第二批特色小镇和全省首批森林小镇，建成幸福美丽新村235个、省级“四好村”99个，晏家坝村获评全国文明村镇。基础设施不断完善，蓉昆高铁进入初设阶段，成资渝高速加快推进，成渝高速高新区站竣工投用，改造国省干道71.6公里、县乡道170公里，新改建通村通社公路500余公里；毗河供水一期工程累计完成总投资的73%、二期工程前期工作完成工程配置编制，城区第三期输水管道加快推进，望水河泄水、鲤鱼水库第二水源、四合水库应急水源等工程前期工作有序开展；川渝第三通道500千伏线路工程等8个电网建设项目竣工投用，城乡宽带网络设施逐步完善。环境治理成效明显，中央环保督察组交办信访件全部办结，348个省环保督察发现问题整改完成328个，其余问题正抓紧整改；河（湖）长制全面落实，市县45条河流“一河一策”管护方案全部编制完成，网箱养鱼全部取缔，规模化畜禽养殖污染整治“三年攻坚”行动全面完成，秸秆禁烧经验全省推广，城区空气质量优良天数303天。市、县（区）城市污水处理厂提标扩能工程和一批园区、乡镇污水处理厂开工建设，医疗废物处置中心投入使用。全域水生态环境建设和大规模绿化全市行动扎实推进，省级森林城市创建有序开展，森林覆盖率达39.8%。

一年来，我们坚持抓改革扩开放，发展动力活力持续增强。重点改革持续深化，供给侧结构性改革扎实推进；深化“放管服”改革，大力推行“一窗进出、并联审批、全程代办”，行政审批提速50%以上，取消行政许可13项、社区证明136项，保留数量为全省最少市州之一；商事制度改革持续推进，新登记企业2500户；农村土地承包经营权确权颁证94%以上，流转土地90万亩；解决融资难、融资贵问题取得积极进展，社会融资成本降低近3亿元；市区公交国有化工作基本完成。开放水平不断提升，主动对接成都“东进”战略，签约制鞋企业400余户、投产70户；积极参加西博会、金博会等重大投资促进活动，成功承办“新兴医疗产业与金融创新”专业论坛，到位国内省外资金315.7亿元；与重庆渝北区、省食药监局等建立战略合作关系；进出口总额11.5亿元、增长31%，到位外资突破1亿美元、居全省第4位。科技创新取得实效，出台“科技创新10条”，新增专精特新企业29户；高新区、县（区）双创孵化平台实现全覆盖，乐至被确定为国家知识产权强县工程试点县；四川现代成功创建省级企业技术中心，中车玉柴、共享铸造、钢管公司设立工程技术研究院（所）。

一年来，我们坚持补短板惠民生，公共服务水平不断提高。脱贫攻坚成效显著，始终把脱贫攻坚作为头等大事，整合各类项目资金11.8亿元，扶贫小额信贷增长5倍，高质量完成80个贫困村退出、4.8万贫困人口脱贫任务，贫困人口义务教育和基本医疗全面保障，往年脱贫对象实现稳定脱贫。民生工程深入实施，省市民生工程、民生实事全面完成，民生支出120亿元，占地方一般公共预算支出66.5%。社会事业协调发展，投入近20亿元改造义务教育学校365所，资阳天立学校启动建设，教育信息化实现“校校通”，增加学位1万余个，“大班额”问题按计划有效化解，办学条件发生可喜变化，30万在校学生受益。国家卫生城市启动创建，公立医院取消药品加成实现全覆盖，分级诊疗取得积极进展。省级文明城市创建扎实推进，首批700个基层文化综合性服务中心全部建成，城乡公共文化体系不断健全。全民健身中心投入使用，市、县（区）均荣获全国群众体育先进单位。城乡社会保障和救助体系更加完善，新增城镇就业1.8万人。民政福利园全面投用。村（居）委会换届工作圆满完成。外事侨台、统计、档案、供销、民族宗教、地方志、气象、慈善、红十字、保密、老龄、残疾人、妇女儿童等工作取得新成绩。

一年来，我们坚持解难题防风险，社会大局保持和谐稳定。矛盾风险有效管控，协调解决120户企业反映的167个问题、82个项目推进中的161个问题；5个政府安置房欠账项目竣工，危旧房棚户区改造3821户；开展政府债务专项清理，建立政府性债务管理办法等4项制度，全面整改违规融资，偿还融资本息15亿元，46.1亿元到期债务全额置换；有序开展互联网金融、非法集资、各类交易所整治，消化不良贷款20.5亿元，不良率下降1.3个百分点。社会治理全面加强，依法治市扎实推进，“七五”普法和“法律七进”深入实施；市本级信访下降26.8%；深化“平安建设”，违法犯罪案件持续下降，圆满完成党的十九大等重大安保维稳任务，乐至荣获全国综治最高荣誉“长安杯”；全面加强防汛减灾、地灾防治、安全生产、食品药品监管工作，有力地保障了人民群众生命财产安全。国防动员、国家安全、“双拥”、应急管理、人防等工作不断加强。

一年来，我们坚持转作风提效能，政府自身建设切实加强。政府运行更加规范，坚决落实市委决策部署，自觉接受人大、政协和社会各界监督，人大代表建议、政协提案办理满意率达100%；修订完善政府工作规则，严格执行重大行政决策法定程序；坚持常务会会前学法制度，严格按照法定权限履行职责、推动发展；政府信息公开和行政权力网上运行力度加大。工作作风持续改进，坚持“两学一做”学习教育常态化，在全市政府系统扎实开展为期1个月的“转作风、提效能、重执行、守规矩”学习教育，查摆整改问题308个，工作作风持续转变；大力推行“互联网+政务服务”，群众办事更加方便快捷。廉政建设不断强化，完善土地出让、专项资金管理、工程招投标等制度，加强审计监督，审减投资6.1亿元；认真履行党风廉政建设“两个责任”，严格落实中央八项规定精神和省市十项规定，深入推进正风肃纪，全面落实公务活动禁酒规定，亲清新型政商关系加速构建，树立了新一届政府良好形象。

各位代表，成绩来之不易，这是省委、省政府和市委坚强领导的结果，是市人大、市政协监督支持的结果，是全市干部群众团结拼搏的结果！在此，我代表市人民政府，向各位代表、政协委员，向各民主党派、工商联、无党派人士，向全市广大干部群众、政法干警、驻资部队官兵、民兵预备役人员，向所有关心支持我市发展的老领导、各界人士，表示衷心的感谢并致以崇高的敬意！

在肯定成绩的同时，我们也清醒地看到存在的问题和挑战：经济发展面临扩量提质双重压力，大项目尤其是产业项目支撑不够；城乡区域发展不平衡，农村基础设施、公共服务比较薄弱；生态环保任务艰巨，社会民生短板还需加快弥补；矛盾风险仍然较多，部分遗留问题处置难度较大；政府自身建设仍需加强，工作质量、效率和水平有待进一步提升。对此，我们一定高度重视，采取有力措施，切实加以解决，不辜负人民群众的期望。

2018年形势与任务

党的十九大作出中国特色社会主义进入新时代的重大政治论断。新时代孕育新希望，我们必须聚焦新要求、抢抓新机遇、落实新部署，奋力开启资阳发展新征程。

新时代新要求。党的十九大提出一系列新思想、新方略、新要求，省委十一届二次全会进一步丰富完善治蜀兴川总体工作格局，市委四届五次全会作出决胜全面小康建设美丽繁荣怡然资阳的部署要求，我们要深刻领会、准确把握，全面贯穿政府工作始终。深刻把握新时代的历史方位。中国特色社会主义进入新时代，是我国发展新的历史方位，我们必须深刻领会新时代的丰富内涵，深刻认识社会主要矛盾的新变化，牢牢把握新时代的历史使命，统筹推进经济社会发展，突出解决发展不平衡不充分问题，在全面建成小康社会的征程中交出圆满的“资阳答卷”。深刻把握新发展理念的要求。我国经济已由高速增长阶段转向高质量发展阶段，我们必须深入贯彻“创新、协调、绿色、开放、共享”新发展理念，加快转变发展方式，积极推动质量变革、效率变革、动力变革，不断增强经济创新力和竞争力，奋力实现更高质量、更有效率、更加公平、更可持续的发展。深刻把握以人民为中心的发展思想。人民对美好生活的向往，是政府的施政取向和奋斗目标，我们必须坚持人民主体地位，坚守民生情怀，切实把增进人民福祉、促进人的全面发展作为一切工作的出发点和落脚点，突出抓重点、补短板、强弱项，着力解决群众最关心最直接最现实的利益问题，努力让群众拥有更多的获得感幸福感安全感。深刻把握新时代资阳发展的总体部署。市委四届五次全会作出加快建设美丽繁荣怡然资阳、发展先进制造业构筑成渝产业高地、加快全域水生态环境建设3个决定，确立“一个愿景、两大战略、三条路径、四个重点、五项工程”总体谋划，这是资阳未来发展的根本指南和行动纲领，我们必须围绕决胜全面小康、建设美丽繁荣怡然资阳宏伟蓝图，不折不扣抓好贯彻落实，奋力推进资阳发展再上新台阶。

新时代新机遇。国家战略给资阳带来重大利好，中央出台新政策推进西部大开发，为我们拓展开放开发空间带来更多机会；深入实施“一带一路”、长江经济带战略，必将推动我市加快建设内陆开放前沿区；加大力度推进成渝城市群建设，有利于我们更好融入成都平原经济区发展；推动经济高质量发展、实施乡村振兴战略，为我市产业转型升级、城乡统筹发展带来更多契机；增加安排地方政府专项债券、专项扶贫资金等，为我们化解政府债务、推进脱贫攻坚带来更多利好。省委省政府重大决策部署惠及资阳，推进天府国际机场和国际空港新城、中国（四川）自由贸易试验区建设，有利于我们高水平建设临空经济区、争创综合保税区；支持资阳发展十条政策措施落地落实，为我市搭建区域合作平台、推进成资一体化提供有力支撑；大力支持中国牙谷建设、轨道交通产业发展，将直接转化为我市发展的强大动力。我市发展站上新起点迎来新格局，经过多年接续奋斗，经济总量突破千亿大关，资阳发展站上新的起点；“7高9轨16快”现代立体综合交通网络加快构建，三个县（区）都处于成渝直线的黄金通道，资阳在成渝经济区中的地位和优势更加凸显；新兴产业风起云涌，制造业向智能化、绿色化、服务化方向加速发展，口腔产业前景广阔，成渝打造万亿级电子信息产业集群，有利于我市加快发展先进制造业，构筑成渝产业高地；城乡面貌、基础设施、生态环境、社会民生持续改善，支撑长远发展的基础更加坚实；更为重要的是，社会大局和谐稳定，干部群众精神奋发，各方智慧力量加速汇聚，资阳蓄势突破、跨越崛起的新格局已经形成！机不可失，时不我待。我们必须牢牢抓住机遇，拼搏实干、砥砺奋进，展现新作为、开创新局面。

新时代新任务。“稳增长”是前提，把稳县域经济、稳投资、稳企业、稳金融、稳就业作为重要抓手，因地制宜做大做强县域特色产业，全面提升县域经济发展水平；大力实施“项目投资攻坚年”活动，坚决扭转投资被动局面；全力支持实体经济发展，切实解决企业困难问题，扩大企业增长面；优化信贷结构，加大对重点项目、小微企业、“三农”等信贷投放；实施积极就业政策，提高就业质量。“提质量”是核心，把高质量发展贯穿经济社会发展各领域、全过程，紧紧围绕质量和效益定目标、出政策、聚资源、上项目，围绕产业转型升级抓改革、求创新、扩开放，加快发展先进制造业、现代服务业和高效种养业，大力培育引进新经济新业态新动能，着力构建现代产业体系，推动经济从量的扩张转向质的提升。“优环境”是保障，创优生活环境，牢固树立“绿水青山就是金山银山”的理念，持续整治污染、改善生态，让资阳天更蓝、地更绿、水更清、生态更美。创优发展硬环境，举全市之力推进成资一体化、中国牙谷和临空产业新城建设，争创国家高新区，构筑产业发展新高地；全力推进轨道交通资阳线、蓉昆高铁、成资渝高速、成资大道等项目建设，更大力度打通“机场通道”“成渝通道”，将资阳区位交通优势转化为经济发展优势；攻坚突破水利瓶颈，扎实推进毗河供水、“张老引水”等工程，全力抓好城乡饮用水源建设和保护，加快推动缺水资阳向水润资阳转变。创优发展软环境，改进服务群众、服务企业的方式方法，建立开明务实的政策体系、便捷高效的政务服务体系、公平公正的法治保障体系。“补短板”是重点，提前两年完成脱贫攻坚任务，全力办好民生工程、民生实事，全面完成大班额化解、教育信息化、医疗卫生服务水平提升等行动计划，加快发展文化体育事业，让人民享有更加幸福安康的生活。“抓统筹”是途径，坚持市县（区）一体、城乡一体，加快创建国家卫生城市、园林城市、全国双拥模范城市和省级文明城市、森林城市，推动城市提品质、小城镇塑特色，建设文明和谐、舒适宜居的美好家园；大力推进乡村振兴，实现全域统筹协调发展。“防风险”是底线，打好风险防范化解攻坚战，严密防控金融风险，碰硬解决政府债务、问题楼盘等矛盾问题，坚决守住安全生产、食品药品安全、社会稳定“三条底线”。这些重点任务，事事关系发展全局、关系长远发展，我们一定扭住不放、久久为功，一个个攻坚，一件件突破，奋力书写资阳发展新篇章。

2018年工作安排

今年是贯彻十九大精神开局之年、改革开放40周年、实施“十三五”规划承上启下的关键一年。政府工作的总体思路是：坚持以习近平新时代中国特色社会主义思想为指导，深入贯彻落实省委、省政府治蜀兴川方略和市委“一个愿景、两大战略、三条路径、四个重点、五项工程”总体谋划，坚持稳中求进工作总基调，以新发展理念为引领，以供给侧结构性改革为主线，以高质量发展为核心，以人民对美好生活的向往为目标，全力做好稳增长、促改革、调结构、惠民生、防风险各项工作，坚决打好“三大攻坚战”，促进经济社会持续健康发展，加快建设美丽繁荣怡然资阳。主要预期目标建议为：地区生产总值增长7.8%，规模以上工业增加值增长9.2%，全社会固定资产投资新口径增长10%，社会消费品零售总额增长11%，地方一般公共预算收入同口径增长8%，城乡居民人均可支配收入分别增长8%、9.5%，城镇新增就业1.45万人，城镇化率提高1.2个百分点。

一、坚定不移实施项目带动，坚决打赢投资翻身仗

加快项目建设。完善项目策划、梯次启动、督促推进等机制，推动市文体中心等150个项目开工建设，临空产业新城基础设施等53个项目加快建设，中国牙谷功能性项目等48个项目竣工投用。抓早抓实前期工作，提高项目成熟度。

扩大民间投资。深化政府与社会资本合作，加快经营型资产股份制改造，推进资产证券化，激活民间投资，提高民间投资比重，力争引入民间资金100亿元。引进股权投资基金，引导企业发行债券、中短期票据，力争实现新三板挂牌零突破。

强化要素保障。大力实施城乡增减挂钩项目，推动更多项目纳入省级重点项目，争取更多用地指标，切实保障项目需求。争取上级财政资金108亿元、政府债券资金40亿元，建好用好40亿元城市发展基金、15亿元产业发展引导基金，力争新增贷款60亿元。加强水、电、气运行调度，保障项目顺利推进。设立1000万元人才引导基金，成立1亿元专项服务基金，引进培育一批能工巧匠和高层次人才。

二、坚定不移推动产业升级，加速培育优势产业

推进工业六大行动。推进制造强市行动，大力发展空铁、齿轨等新制式轨道交通装备制造，加快轨道交通产业园建设，做大做强商用车、专用车整车生产，推动零部件配套企业集群发展；全力发展口腔装备材料产业，口腔企业达到60户；提质发展食品饮料、医药产业，加快发展电子信息及大数据产业，先进制造业占比达38%。推进项目带动行动，确保安井食品等15个项目竣工投产，中石油（安岳）天然气脱硫厂等4个项目加快建设，万贯鞋业产业园二期等22个项目开工建设；加大工业项目引进力度，积极发展服务型制造和生产性服务业。推进创新引领行动，引导企业加大科技研发投入，筹建四川大学国家技术转移分中心和产业技术研究院，新增一批创新平台；实施新能源机车创新研制等13个创新项目，完成创新投入6亿元；新发展国家高新技术企业5户、科技型企业20户，高新产值突破410亿元。推进园区提质行动，完善园区基础设施及功能配套，创新园区运营模式，新入园企业50户以上，提升园区生产能力、就业能力、纳税能力。推进企业培育行动，推动一批重点企业建立现代企业制度，做大做强做优一批骨干企业，力争亿元企业达135户、10亿元企业达28户，新培育规模企业20户以上；培育企业家队伍，引导企业家专注实业、创新创造、追求卓越，培育保护企业家精神和工匠精神。推进质量提升行动，实施标准引领、品牌带动、质量基础、质量安全工程，提高“资阳造”产品品质和竞争力，确保省级质量强市示范城市通过验收。

实施服务业五大计划。实施项目建设计划，加快龙飞物流基地等179个服务业项目建设，确保中韩国际城市综合体等52个项目竣工投用。实施企业成长计划，着力培育资阳万达等30个服务业企业，打造30个省级以上服务品牌，新增亿元服务业企业10户。实施产业发展计划，培育电商交易额超千万企业20户，新增小微电商企业200户；加快推进3个县（区）物流园区建设，推动格罗唯视争创4A级物流企业；加快发展健康养老产业；全面完成国家级旅游业改革创新先行区建设，实施“绿色资阳”旅游行动计划，旅游收入185亿元。实施消费刺激计划，加快汽车、鞋材、农副产品交易等专业市场建设，打造区域性商贸中心，大力开展“资阳产品全国行”等市场拓展活动，办好柠檬节等特色节会，扩大市场消费。实施改革创新计划，积极申创综合保税区，深度参与中国（四川）自由贸易试验区建设。

抓实农业五大重点。大力创品牌，推广运用“互联网+农业”模式，加大安岳柠檬、雁江蜜柑、乐至黑山羊等特色农产品推介营销力度，新申报“三品一标”农产品20个，提升“资味”品牌影响力。着力强技术，支持科研院所建立农业试验示范基地，加快农产品标准体系建设，加强新品种、新技术、新设备推广使用。加快建基地，全面落实粮食安全、“菜篮子”责任制；推进特色产业提质扩面，建设种养循环园区30个，推动安岳创建国家现代农业产业园、乐至创建国家畜牧业绿色发展示范县；积极发展观光农业、休闲农业、都市农业等新产业新业态，建设成渝绿色农产品供应基地和乡村旅游目的地。突出搞加工，立足特色农产品资源，加快建设中和、龙台、中天3个农产品加工园区，大力发展精深加工，提高农产品附加值。深入抓改革，深化农村产权制度改革，发展农村集体经济，吸引工商资本、金融资本、社会资本投入农业农村；新培育一批省级专合组织、家庭农场。

三、坚定不移推进城乡统筹，着力提升宜居水平

着力提高规划水平。强化规划引领，编制城市建筑风貌、人防工程、慢行系统、绿道等专项规划，全面完成临空经济区总规、控规编制。加强城市设计，对城市核心区、干道两侧、江河两岸开展专门设计，打造城市景观和滨水景观。严格规划执行，完成城镇建筑风貌管理立法，维护规划的权威性和严肃性。

攻坚推进城市建设。大力推进城市“双修”，持续实施“六大工程”，完善城市功能，提升城市品质。实施“改旧扩新”工程，强力推进城北片区改造、城南大道和外环路提升，改造棚户区项目13个、3510户；扎实抓好临空经济区骨干道路、公共配套和产业园区路网等21个项目建设，实现首批道路主体基本成型，产业导入快速破题；持续推进城东经七路、黄泥河生态景观长廊等项目建设，促进“一城四区”同步提升。实施“疏脉通络”工程，扎实推进娇子大道西延线、沱西滨江路等项目，打通建设南路西延线等4条断头路，加快推进雷音大桥前期工作，整治159条（处）背街小巷，完善地下管网设施，畅通城市骨干路网，疏通城市毛细血管。实施“风貌塑造”工程，整治城区沿街风貌，完成建设北路、滨河西路等道路“七位一体”改造，陆续实施第二批城区95条道路改造，提升城市风貌。实施“增绿扮靓”工程，开工建设天台山公园、康乐公园、白沙坝郊野公园和沱江生态修复项目，建成城市水体公园，美化九曲河、沱江城区段滨水岸线，大力开展城区闲置空地补绿和裸露山体植被修复，彰显山水特色，打造城市绿肺。实施“文化筑魂”工程，逐步恢复“雁江古渡”“宝刹晨钟”等雁城八景乡愁记忆，启动建设一批公共文化服务体系项目，塑造资阳历史记忆和时代特色。实施“提质惠民”工程，大力推进“厕所革命”，全市新改扩建187座厕所，规划实施一批集贸市场、停车场等便民项目，建成安置房134万平方米，引进房地产领军企业开发精品楼盘，推动房地产、建筑业提质发展，加强城市防护设施建设，让群众住得更加舒适、更加安全。

切实加强城市管理。实施精细化管理，全面落实“街长制”，强化网格化管理，打造市容市貌示范街（区）；实施规范化管理，建立健全城市长效管理机制，持续整治车辆乱停乱放、乱搭乱建和环境卫生死角；实施法治化管理，坚持严格执法、公正执法、文明执法，提高城管执法能力和水平；实施智慧化管理，加快建设“智慧城管”平台，实现全方位覆盖、全时段监管、高效能运转，全力营造“畅洁绿美、规范有序”的城市环境。

统筹抓好城镇建设。推动安岳县城“东拓南延”发展，抓好海绵城市建设试点项目；支持乐至县建设生态宜居园林城市，大力实施市民500米休闲计划。深化拓展“百镇建设行动”，加快打造牙谷、柠檬等一批特色小镇，力争创建2个国家级特色小镇。

大力推进乡村振兴。实施农村产业提质、美丽乡村建设、乡村治理提升“三大行动”，建好“四好农村路”，加快提升农业基础设施、产业发展、公共服务，构建城乡一体发展体制机制。加快推进农村人居环境整治，重点加强垃圾处理、污水治理、土坯房改造，把农村妆扮得更加靓丽、让农民生活得更加舒心。建成幸福美丽新村200个，创建省级“四好村”90个、市级200个。

四、坚定不移加强基础建设，持续改善发展条件

大力推进交通建设。加快建设成资渝高速，全力推动蓉昆高铁等重点项目，抓好绵遂资内宜铁路等项目前期工作，加密畅通成渝的骨干通道。开工建设资安快速通道、成渝高铁资阳东汽车客运站，加快建设陈毅故居旅游大道，改造县乡道100公里，新改建村社道300公里，提高市域交通畅达水平。优化城市公交线路和站点布局，提高公共交通运行效率。

强力推进水利建设。确保毗河供水一期工程累计完成投资58.7亿元，全力争取二期工程调整纳入国家“十三五”水利规划，完成可研编制并力争完成审批，建成关刀桥水库。开工建设望水河泄水工程，力争开工鲤鱼水库第二水源、四合水库应急水源工程，加快推进安岳城西水库、乐至宝石水库前期工作。

持续改善能源通信设施。加快推进220千伏、110千伏输变电工程建设，实施农网改造升级工程，提高农村电网安全性可靠性。新建4G通信基站800个，推动农村光纤覆盖率达90%以上。抓好天然气开发利用，新勘探天然气井20口。

五、坚定不移推进绿色发展，切实保护生态环境

重拳整治污染。坚决整改突出问题，持续整治中央环保督察反馈问题、省环保督察发现问题，巩固整改成果，确保问题不反弹。持续打好污染防治“三大战役”，加强大气污染防治，确保城区空气质量优良天数达80%以上；深入落实河（湖）长制，大力实施“清河、护岸、净水、保水”行动，持续改善水环境质量；加强农业投入品减量控害，强化危险废物管理，为子孙后代留下更多沃土良田。强力推进环保设施建设，开工市垃圾环保发电项目，加强城乡垃圾收集处理设施建设；加快推进市、县（区）城市生活污水处理厂提标扩能、市第二水厂扩建工程，建成雁江中和、乐至工业园污水处理厂，整体推进89个乡镇污水处理厂建设。加大环保执法力度，发动全社会力量共抓环境保护，严肃查处违规排放、超标排放行为，让市民饮用到更洁净的水、呼吸到更清新的空气。

提高生态质量。抓好全域水生态环境建设，积极申报山水林田湖草生态保护修复项目，开工建设一批水生态项目，规划建设一批湿地公园。实施大规模绿化全市行动，完成营造林11万亩，新增森林面积8.7万亩，森林覆盖率提高到40.5%。着力发展绿色低碳产业，严格项目环境准入，鼓励企业使用清洁能源，大力发展循环经济，减少污染排放；积极倡导低碳生活、绿色出行。

六、坚定不移推进改革开放，充分释放发展活力

全力推进成资一体化。主动对接成都“东进”战略，积极承接非省会城市功能疏解，全力推动轨道交通资阳线、成资大道、“张老引水”等合作项目开工建设，引进更多产业转移企业入驻、建设和投产，实现基础建设、产业发展、公共服务等五个方面的更大突破。

全面深化各项改革。推进投融资体制改革，实施投资项目“审批不见面”改革，规范企业投资项目监督管理；积极引导更多社会资本参与公共服务项目建设。深化财税体制改革，强化税收征管，完善事权和支出责任相适应的财政制度，健全完善市本级与雁江区、高新区、临空经济区财税管理体制。加快国有平台公司实体化转型，理清职能职责划分，压实平台公司全面承担重点项目融资、建设和营运中的主体责任。建立现代医院管理制度，进一步理顺政府整合开放办医体制，完善医疗、医保、医药联动机制。深化国有企业、金融、价格、生态文明、教育、文化、供销、安全生产等领域改革。

着力推动招强引优。突出产业招商，务实开展委托招商、以企招商、专业招商，大力实施“分层分类、定点定向”精准招商，精心组织参加西博会等重大投资促进活动，引进一批龙头型、补链型优质企业，到位国内省外资金320亿元。

深化拓展区域合作。组织参加“万企出国门”“资阳企业丝路行”等活动，实现进出口总额9.5亿元，到位外资5000万美元。深化与省级部门、央企省企、科研院所、高等学校等的战略合作，推动更多合作项目落地见效。

七、坚定不移实施脱贫攻坚，持续改善社会民生

决战决胜脱贫攻坚。今年是集中攻坚的最后一年，要聚焦“两不愁、三保障”和“四个好”目标，下足“绣花”功夫，推进精准扶贫、精准脱贫，确保81个贫困村退出、4.4万贫困人口脱贫；深入开展“回头看”“回头帮”，确保已脱贫对象稳定脱贫。

全力办好民生实事。集中力量办好省市民生工程和民生实事。全面实施全民参保计划，完善城乡统一的医疗保险和大病保险制度，让发展成果惠及全市人民。开工建设市民中心、雁江区殡仪馆迁建工程，建成市老干部活动中心（老年大学），推动市、县（区）残疾人康复中心全部投用。

全面发展社会事业。促进教育优先发展，深化义务教育均衡发展工作，确保通过国家审核认定；加快教育信息化建设，基本实现城乡学校“班班通”；加快实施幸福博文学校、乐至博骏公学建设和安岳实验中学迁建等项目，推动资阳天立学校年内招生，完成天府口腔医学职业技术学院主体工程，再建一批公办幼儿园，完成“大班额”化解规划任务；加强师德师风建设，培育一批好学校、好校长、好老师。全面推进“健康资阳”建设，持续深化与省人民医院、华西医院、华西附二院等省部级龙头医院的合作，推动市中医院由成都中医药大学直管，建成“三甲”医院，成为成都中医药大学附属医院，努力将资阳合作办医模式打造成全省医联体典范；培育名医、名科、名院，建成1个国家级、2个省级重点专科，支持中医药发展，构建城乡优质医疗服务网，全力打造成都周边区域医疗中心；打好国家卫生城市创建攻坚战，确保通过省上复审。加快发展文体事业，完成市博物馆主体工程，再建成700个基层文化综合性服务中心，策划推动安岳石刻博物馆等项目，精心打造《陈毅回川》《千年普州》等精品力作，进一步创响“蜀人原乡”文化品牌；广泛开展全民健身活动，积极备战第十三届省运会。培育和践行社会主义核心价值观，倡导健康文明生活方式，提高市民道德修养和文明素质。完成首部《资阳市志》编撰。

加强外事侨台、统计、档案、供销、民族宗教、气象、慈善、红十字、保密、老龄、残疾人、妇女儿童等工作。

八、坚定不移创新社会治理，全力维护和谐稳定

严密防范各类风险。深入开展“走企业、解难题”活动，积极帮助困难企业摆脱困境、渡过难关。完善金融监管体系，加大“处非”力度，开展互联网金融专项整治，严厉打击恶意逃废银行债务行为，推动不良贷款持续“双降”，加快建设金融生态示范市。以更坚定的决心、更严格的措施，完善政府债务规范化管理制度，加强PPP项目和政府购买服务项目协调管控，科学调控政府中长期支出，健全债务风险防控机制，严肃问责追究，确保债务风险总体可控。持续用力处置毛地拍卖、问题楼盘等遗留问题。

全力维护稳定安全。加强和改进信访工作，深入推进依法治访，集中攻坚化解一批信访积案难案。深化立体化治安防控体系建设，建成“天网”三期工程，依法严厉打击各类违法犯罪活动。完善综合应急管理机制，提高应急处突能力。加强安全生产和食品药品安全监管，坚决防范和杜绝重特大安全事故、有影响的食品药品安全事故。

加强民主法治建设。健全基层治理体系，增强社区治理能力。深入开展“七五”普法和“法律七进”，加强法律服务和法律援助，提高依法治市水平。推进社会诚信建设，建立健全守信激励、失信惩戒机制。加强国家安全教育和国防动员能力建设。

九、坚定不移抓好自身建设，加快打造人民满意政府

提高政治站位。牢固树立“四个意识”，坚决维护以习近平同志为核心的党中央权威和集中统一领导，始终在思想上政治上行动上同党中央保持高度一致。坚决服从市委领导，自觉接受人大、政协及社会各界监督，高质量办理人大代表建议和政协提案，认真听取各民主党派、工商联、无党派人士、各人民团体的意见，调动一切积极因素，凝聚新时代资阳发展的磅礴力量。

加强学习创新。学习新理念新知识，拓展眼界和视野，跟上知识更新节奏和时代前进步伐，增强适应新时代、完成新任务的本领。始终保持敢闯敢试、锐意进取的精神面貌，解放思想、大胆探索、勇于创新，用新理念、新思路、新办法化解矛盾问题、推动加快发展。

优化政务环境。开展“政务服务效能提升年”活动，全面完成“两集中、两到位”，指导帮助企业和群众完善审批要件，提高审批效率，努力让企业感受到办事方便、服务优质和包容尊重。加强基层便民服务平台建设，着力打通政策落实、为民服务“最后一公里”。

严格依法行政。坚持重大行政决策法定程序，加强社会稳定风险评估，扩大公众参与度，提高依法科学民主决策水平。坚持依法行政，推进政府立法，运用法治思维和法治方式解决问题、推动发展。坚持公正文明执法，做好“行政执法与刑事司法”衔接工作，加强行政复议，规范行政行为，切实提高政府公信力。

持续改进作风。扎实开展“不忘初心、牢记使命”主题教育活动，大力弘扬“红船”精神，持续深化政府系统作风建设，坚决纠正形式主义、官僚主义。大力发扬“钉钉子”精神，主动作为，勇于担当，增强敢闯敢试的干劲拼劲，切实提高执行力、落实力，确保政令畅通令行禁止。牢固树立精品意识、效率意识，以全国、全省先进典型为榜样，扎实开展“对标行动”，推动政府工作快节奏、高标准落实。强化目标绩效管理、督查督办和问责追究，向“庸懒散浮拖”“为官不为”等行为宣战，宣传正面典型，弘扬新风正气，查处曝光负面典型，绝不姑息。

深化廉政建设。全面履行党风廉政建设责任，加强对政府系统干部的教育、管理和监督。严格落实中央“八项规定”精神和省市“十项规定”实施细则，坚决反对铺张浪费。深化政府系统纪律建设，强化审计监督，严查违纪违法行为，树立廉洁政府形象。

各位代表！资阳进入新时代，发展开启新征程。让我们在习近平新时代中国特色社会主义思想的指导下，在省委、省政府和市委的坚强领导下，不忘初心、牢记使命，锐意进取、扎实工作，为推动经济社会发展再上新台阶，加快建设美丽繁荣怡然资阳而努力奋斗！
